# Catalytic competence, structure and stability of the cancer-associated R139W variant of the human NAD(P)H: quinone oxidoreductase 1 (NQO1)

**DOI:** 10.1111/febs.14051

**Published:** 2017-03-17

**Authors:** Wolf-Dieter Lienhart, Emilia Strandback, Venugopal Gudipati, Karin Koch, Alexandra Binter, Michael K. Uhl, David M. Rantasa, Benjamin Bourgeois, Tobias Madl, Klaus Zangger, Karl Gruber, Peter Macheroux

**Affiliations:** 1 Institute of Biochemistry, Graz University of Technology, Austria; 2 Institute of Molecular Biosciences, University of Graz, Austria; 3 Institute of Molecular Biology and Biochemistry, Medical University of Graz, Austria; 4 Institute of Chemistry, University of Graz, Austria

**Keywords:** cancer, crystal structure, enzyme kinetics, microcalorimetry, NMR-spectroscopy, single nucleotide polymorphism

## Abstract

The human NAD(P)H:quinone oxidoreductase 1 (NQO1; EC 1.6.99.2) is an essential enzyme in the antioxidant defence system. Furthermore, NQO1 protects tumour suppressors like p53, p33^ING1b^ and p73 from proteasomal degradation. The activity of NQO1 is also exploited in chemotherapy for the activation of quinone-based treatments. Various single nucleotide polymorphisms are known, such as NQO1*2 and NQO1*3 yielding protein variants of NQO1 with single amino acid replacements, i.e. P187S and R139W, respectively. While the former NOQ1 variant is linked to a higher risk for specific kinds of cancer, the role, if any, of the arginine 139 to tryptophan exchange in disease development remains obscure. On the other hand, mitomycin C-resistant human colon cancer cells were shown to harbour the NQO1*3 variant resulting in substantially reduced enzymatic activity. However, the molecular cause for this decrease remains unclear. In order to resolve this issue, recombinant NQO1 R139W has been characterized biochemically and structurally. In this report, we show by X-ray crystallography and 2D-NMR spectroscopy that this variant adopts the same structure both in the crystal as well as in solution. Furthermore, the kinetic parameters obtained for the variant are similar to those reported for the wild-type protein. Similarly, thermostability of the variant was only slightly affected by the amino acid replacement. Therefore, we conclude that the previously reported effects in human cancer cells cannot be attributed to protein stability or enzyme activity. Instead, it appears that loss of exon 4 during maturation of a large fraction of pre-mRNA is the major reason of the observed lack of enzyme activity and hence reduced activation of quinone-based chemotherapeutics.

## Introduction

NAD(P)H:quinone oxidoreductase 1 (NQO1; EC 1.6. 99.2) [1] is an important enzyme in the human antioxidant defence system. Among other functions, the dimeric flavoprotein is catalysing the conversion of quinones to hydroquinones preventing the formation of semiquinone radicals [2]. Yet, another important role is the regulation and stabilization of various tumour suppressors like p33^ING1b^, p53 and p73. This effect appears to be related to the interaction of NQO1 with the 20S proteasome in a NADH-dependent manner [3,4]. Single nucleotide polymorphisms result in the expression of different protein variants of NQO1. The two most prevalent variants in the human population are NQO1*2 (*NQO1 609C>T*; NQO1 P187S; allelic frequency: 0.22–0.47) and NQO1*3 (*NQO1 465C>T*; NQO1 R139W; allelic frequency: 0.00–0.05), which are connected to a higher risk for specific cancers [5–11]. Several studies have focused on NQO1*2 and have shown a reduction or even a loss of the enzymatic activity of NQO1 P187S [12–14]. Furthermore, this single nucleotide polymorphism (SNP) gives rise to reduced stability of the protein and to a loss of the FAD cofactor. On the other hand, the involvement of NQO1*3 in the development of cancer is currently unclear. Initial observations indicated that splicing of the transcript of NQO1*3 yields mature mRNA lacking exon 4, which consequently leads to the loss of the FAD binding domain [15]. In the mitomycin C-resistant tumour cell lines, HCT 116-R30A solely the mRNA of NQO1*3 could be detected while in the mitomycin C sensitive HCT 116 cell line mRNAs of NQO1*1 and NQO1*3 were detectable [16]. These findings led to the assumption that the higher cancer risk for the NQO1*3 polymorphism might be caused by erroneous splicing of the pre-mRNA derived from NQO1*3. As a matter of fact, the nucleotide transition found in NQO1*3 disrupts the consensus sequence of the 5′ splicing site required for the correct splicing by the spliceosome and thus rationalizes the observations mentioned above [11]. Since the full length mRNA of NQO1*3 is still representing one to two-thirds of the whole mRNA [11], it is unclear if the higher risk for specific cancers can be explained solely by erroneous splicing. Thus far enzyme activities were determined only in cell extracts [11] or with the unspecific redox dye 2,6-dichloroindo-phenol (DCPIP) [17] but not with a quinone substrate. Moreover, information concerning the potential impact of the R139W exchange on structural properties of the enzyme is currently not available.

A loss of enzymatic activity is increasing the toxicity of benzene as well as aggravating the cancer treatment of patients [18]. The broad substrate specificity of NQO1 allows the activation of chemotherapeutic prodrugs, like mitomycin C or β-lapachone. Since various tumours are upregulating the NQO1 levels, these chemotherapeutics are acting more specific on cancer than healthy cells [19–21]. The success of the prevalent cancer treatment with cisplatin is also affected by the NQO1 activity. One limitation for the use of cisplatin is the induced nephrotoxicity. Activation of NQO1 can improve the negative effects of the treatment to the kidneys while a loss of enzyme activity can cause an accelerated damage of the renal system [22]. Taken together the status of NQO1 expression and activity is essential for the success of quinone-based chemotherapies, and therefore detailed biochemical and structural studies are paramount to generate a sound basis for the development and design of cancer intervention strategies.

In order to remedy the current lack of sound biochemical information on the NQO1 R139W variant, we have undertaken a biochemical, enzymatic and structural investigation to fully comprehend the effect of this widely occurring variant of the human enzyme.

## Results

### Expression and basic biochemical characterisation of the R139W variant

**Fig. 1:**
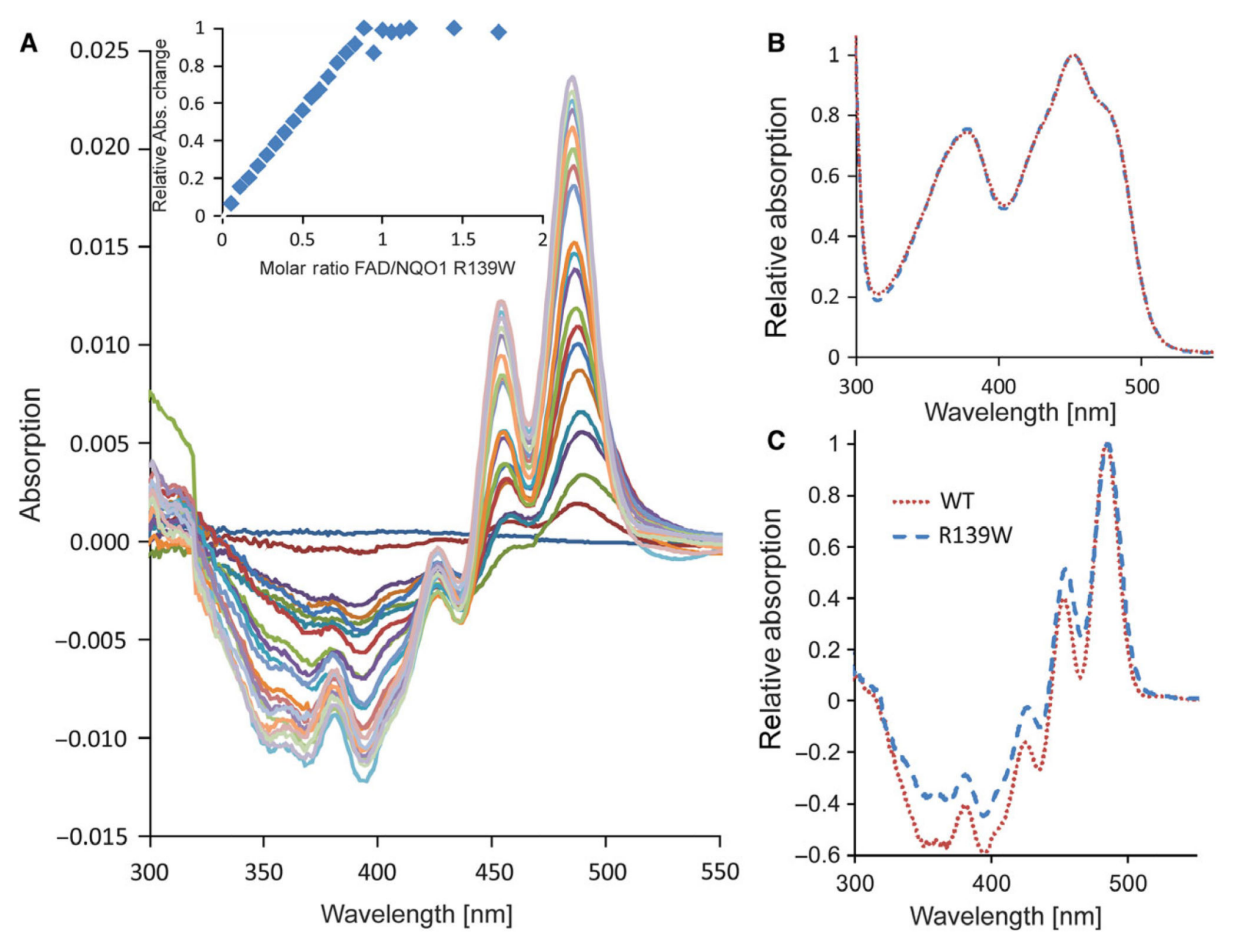
UV-visible and difference titration absorption spectra of wild-type NQO1 and NQO1 R139W. (A) Difference titration spectra of 800 μL NQO1 R139W (45 μm) with 0–42 μL in 2 μL intervals FAD (1 mm). The insert shows the change of the absolute absorption values at 436 nm and 455 nm against the FAD/protein molar ratio with two additional points (with additional 10 μL FAD each) compared to the main figure. (B) Absorption spectra of wild-type NQO1 and NQO1 R139W normalized to the maximum at 450 nm. (C) Difference titration spectra of wild-type NQO1 and NQO1 R139W with protein/FAD ratio of 1 normalized to the maximum at 480 nm.

Heterologous expression of the NQO1 R139W variant in *Escherichia coli* BL21 yielded similar amounts of soluble protein as was previously reported for wild-type NQO1 [14]. Preparations of the R139W variant showed the typical yellow colour indicating that the protein tightly binds the FAD cofactor in stark contrast to the P187S variant that was isolated largely as an apoprotein [14]. Further analysis showed that wild-type NQO1 and the R139W variant have nearly identical absorption spectra with maxima at 375 and 450 nm (Fig. 1). In addition, titration of apoproteins with FAD gave rise to a similar difference absorption spectrum indicating that the FAD binding pockets provided by wild-type NQO1 and the R139W variant are comparable (Fig. 1).

The melting points of the NQO1 WT and NQO1 R139W variant were determined in different buffers and showed a decrease of 2 °C for the R139W variant. Measurements with SYPRO^®^ Orange of the holo- and apo-form of the proteins showed a decrease in the melting points for the apoprotein. The small differences in the melting points observed between FAD and SYPRO^®^ Orange may indicate that the latter has an adverse effect of thermal stability by promoting the unfolding of the protein (Table 1).

### Isothermal titration calorimetry and small-angle X-ray scattering

**Fig. 2:**
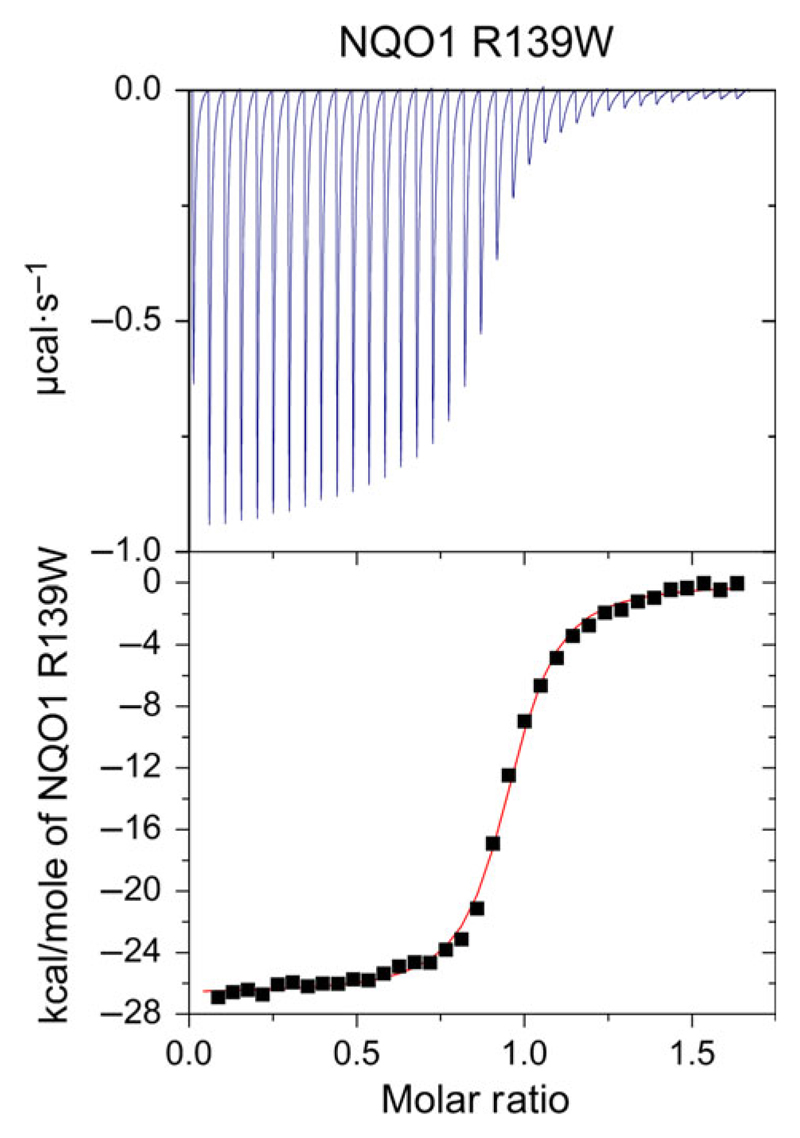
Isothermal titration microcalorimetry measurement of NQO1 R139W. NQO1 R139W: 35 injections with 6 μL of 298 μm NQO1 R139W apoprotein solution in 29 μm FAD solution and 300 s spacing (top) at 25 °C. From three independent measurements under the same conditions, the dissociation constant was calculated to *K*_D_ = 155 ± 27 nm. Data was fitted using the one binding site model.

To obtain quantitative information on the binding affinity of the FAD cofactor to the R139W, variant isothermal titration calorimetry experiments were conducted. As reported recently, reproducible measurements are obtained by titration of a fixed concentration of FAD with apoprotein [14]. The raw data could be nicely fitted to a one binding site model [14] (Fig. 2). The average of three measurements was used to determine the *K*_D_ values for the NQO1 R139W variant as 155 ± 27 nm, which is 2.5-fold higher than the *K*_D_ for wild-type enzyme [14]. Thus, it can be concluded that the arginine to tryptophan replacement has only a marginal effect on the binding affinity of the FAD cofactor.

**Fig. 3:**
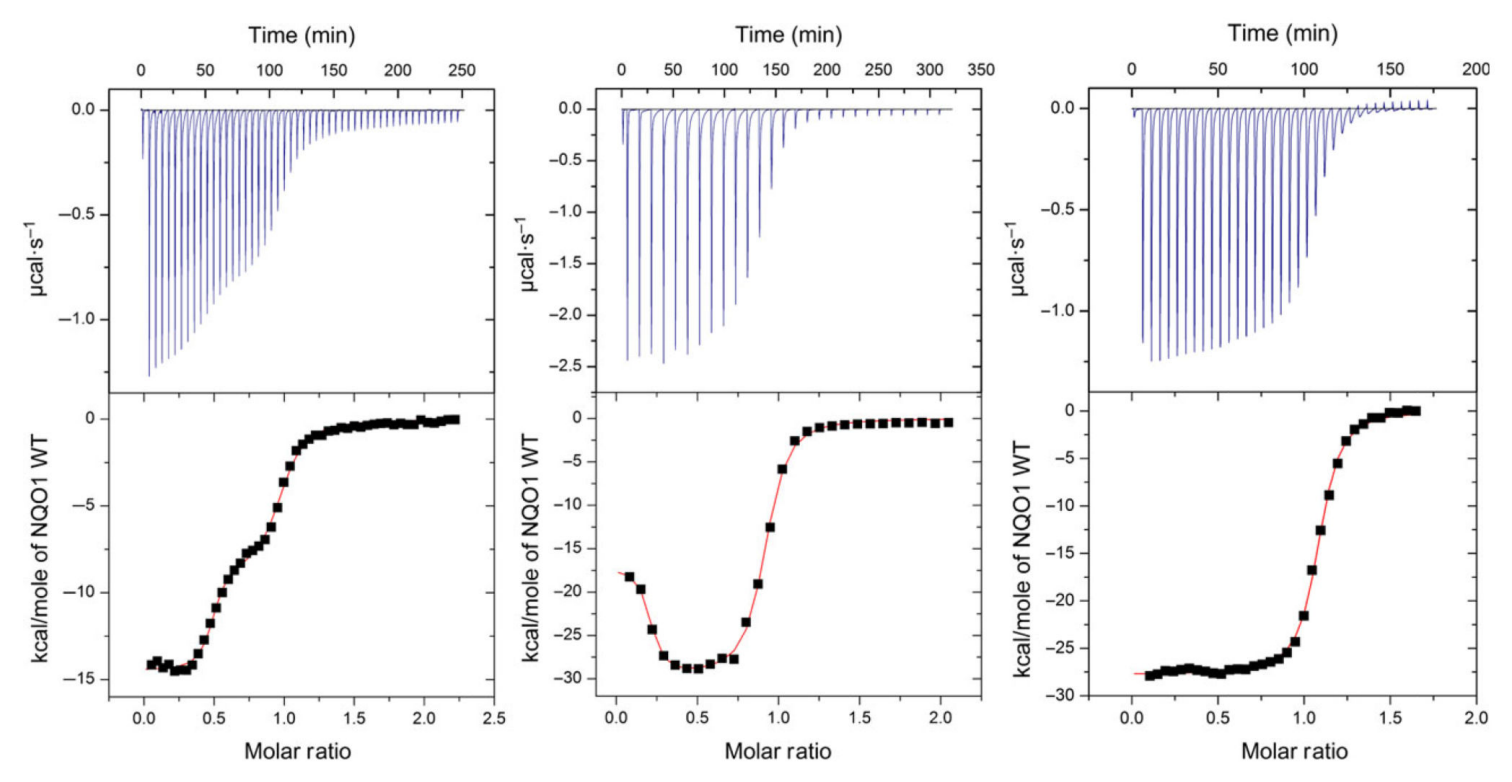
Isothermal titration microcalorimetry measurements of NQO1 wild-type in HEPES buffer. The left and the middle measurements were conducted with apo-NQO1 in the sample cell of the microcalorimeter and FAD in the injection syringe. Data was fitted using the two binding site model. Left: First injection with 2 μL and 49 injections with 6 μL of 477 μm FAD solution in 49.4 μm NQO1 apoprotein solution and 300 s spacing at 25 °C. The determined *K*_D_ and *N* values are: *K*_D1_ = 9.2 nm; *K*_D2_ = 780 nm; N1 = 0.47; N2 = 0.5. Middle: First injection with 2 μL and 27 injections with 10 μL of 457 μm FAD solution in 46.8 μm NQO1 apoprotein solution and 600 s spacing at 25 °C. The determined *K*_D_ and *N* values are: *K*_D1_ = 4 nm; *K*_D2_ = 295 nm; N1 = 0.18; N2 = 0.71. The right measurement was conducted with FAD in the sample cell of the microcalorimeter and apo-NQO1 in the injection syringe. Data was fitted using the one binding site model. First injection with 2 μL and 34 injections with 6 μL of 284 μm NQO1 apoprotein solution in 29 μm FAD solution and 300 s spacing at 25 °C. From three independent measurements under the same conditions, the dissociation constant was calculated to *K*_D_ = 64 ± 23 nm.

**Fig. 4:**
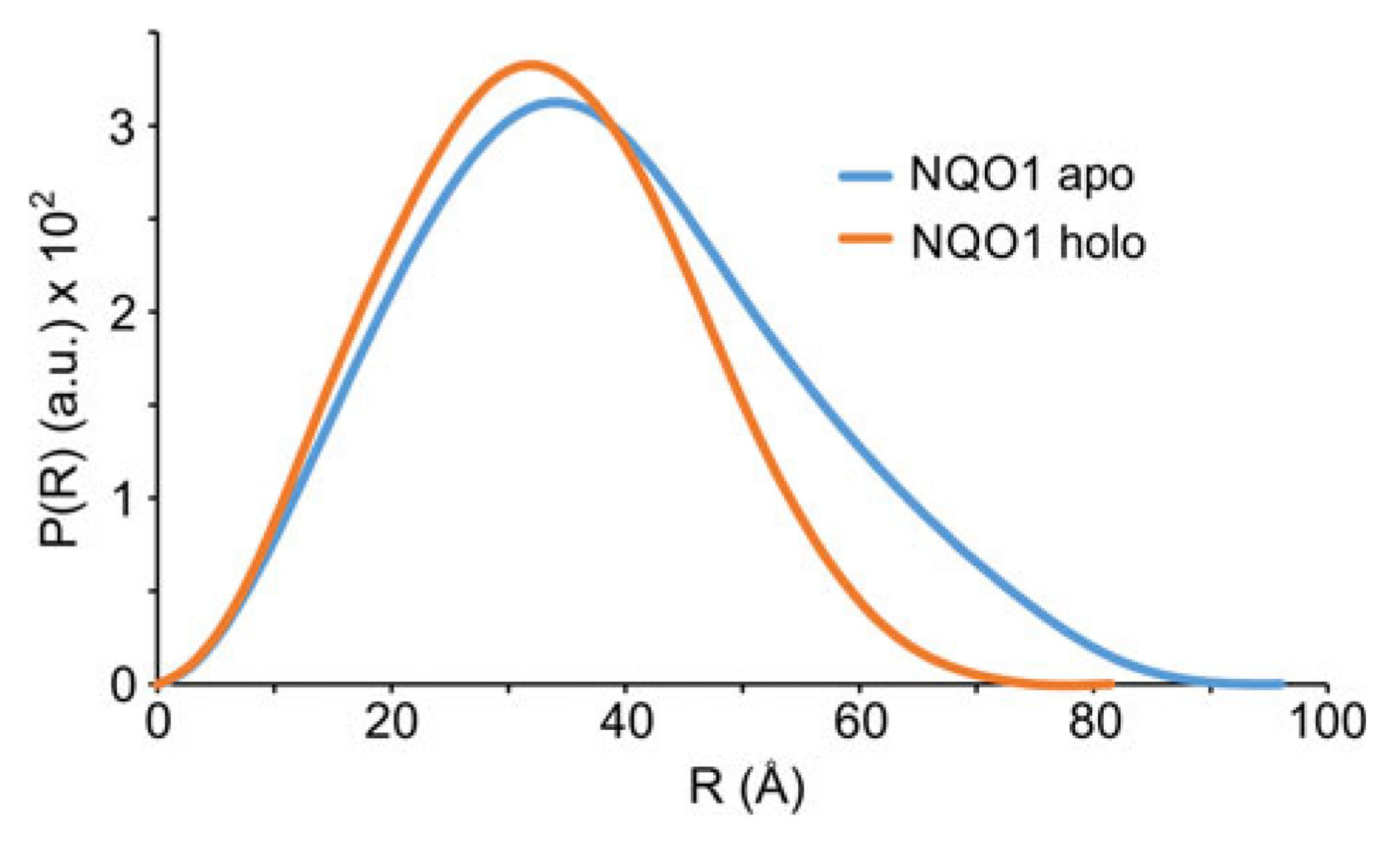
Small-angle X-ray scattering measurement of NQO1. SAXS data showing a comparison of the experimental radial density distribution (P(r)) of apo- and holo-NQO1 in cyan and orange, respectively.

In this context, it is important to note that the experimental setup of the isothermal titration microcalorimetry (ITC) experiment is critical to obtain reliable data both in terms of stoichiometry and the dissociation constant. In principal, reversal of the order of titration should not influence the outcome of the experiment in terms of the model used to fit the raw data. However, when apoproteins (wild-type as well as the R139W variant) were titrated with FAD the raw data could not be satisfactorily fitted with a one binding site model. Instead, the raw data were best fitted to a two binding site model, a result that is difficult to reconcile with the structural identity of the two FAD binding pockets in the homodimeric protein (Fig. 3; data with wild-type). Interestingly, we observed that variable amounts of protein had apparently precipitated during the experiment. Thus, a possible source for the irreproducibility observed with this particular experimental setup appears to be the instability of apoproteins in the microcalorimeter cell where constant stirring is required over the entire time course of the experiment. Similarly, when wild-type NQO1 was constantly stirred in an optical cuvette, we observed a gradual increase at 600 nm indicating denaturation and finally precipitation of protein. After removal of the precipitated protein by centrifugation, the residual protein appeared to be intact as it behaved similar to unstirred protein in size exclusion chromatography. Attempts to stabilize the apoproteins by lowering the temperature (e.g. 4 °C) or testing different buffers influenced the overall shape of the obtained raw data but failed to improve the reproducibility of the experiment. Importantly, SAXS measurements of wild-type NQO1 showed that the protein forms a dimer in solution both in the holo- and the apo-form, with the holoprotein being more compact compared to the more extended apoprotein (radius of gyration 2.5 and 2.94 nm, Fig. 4). Thus, the dimeric apoprotein seems to be partially open or unfolded as a consequence of FAD depletion.

### Kinetic measurements

The reductive rates for the NQO1 R139W variant, with NADH and NADPH as reducing cosubstrates, were determined. As shown in Table 2, the limiting values for reduction are comparable to those determined earlier for wild-type NQO1 [14]. Interestingly, the observed transients showed a biphasic behaviour with a second slower substrate-independent rate, which might have been caused by product release. The oxidative half reaction of the NQO1 R139W variant was completed within the dead time of the stopped flow device as was reported previously for the wild-type and the NQO1 P187S variant [14].

**Fig. 5:**
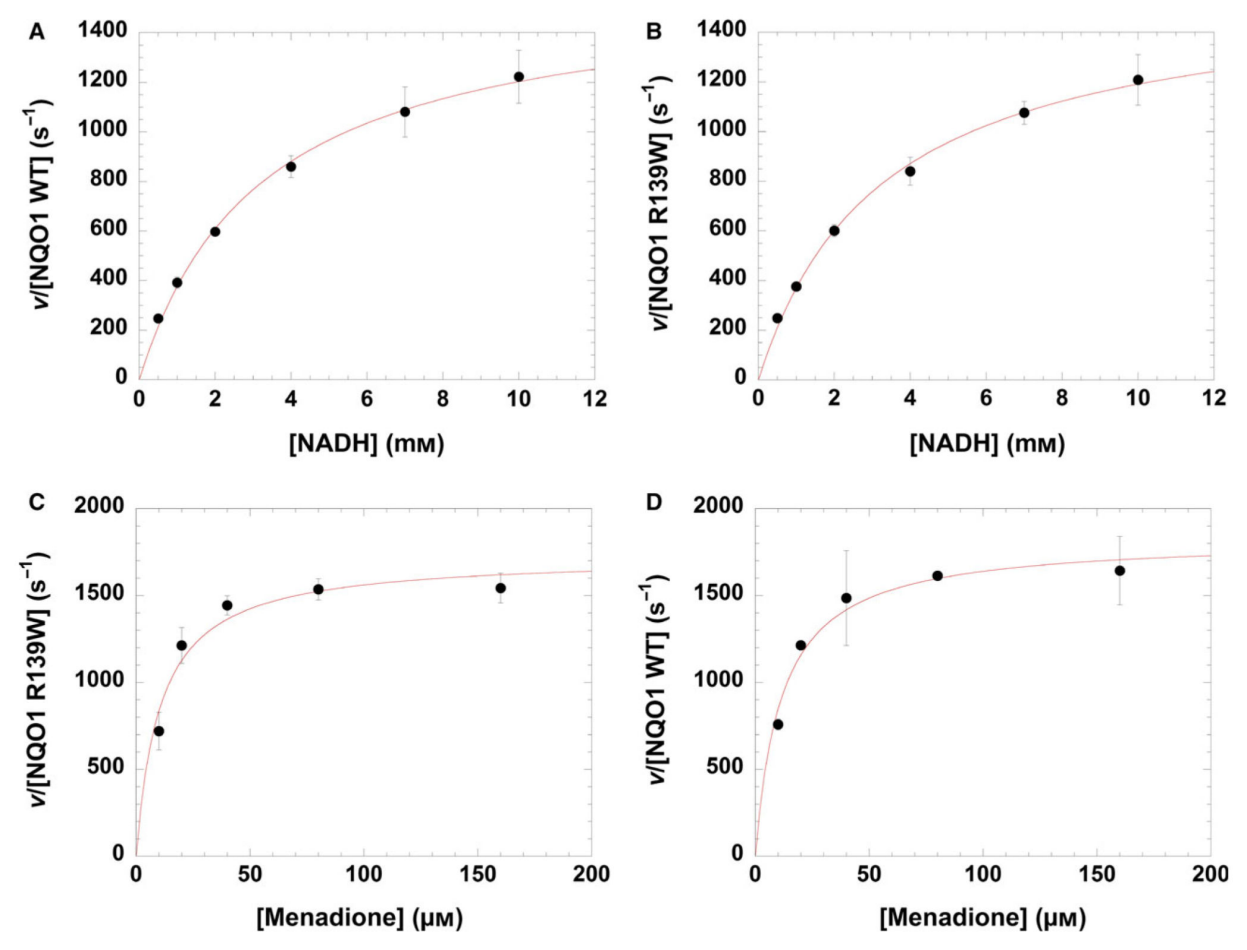
Steady state kinetics of NQO1 WT and NQO1 R139W. In the case of variation of NADH, the velocity *v* over the enzyme concentration is plotted against NADH concentration for NQO1 WT (A) and NQO1 R139W (B). For the variation of menadione, the velocity *v* over the enzyme concentration is plotted against menadione concentration for NQO1 WT (C) and NQO1 R139W (D). Standard deviations are shown with error bars.

In addition, we have determined steady state kinetic parameters for WT enzyme and the R139W variant using NADH and menadione as reducing and oxidizing substrate, respectively. As summarized in Table 3 and seen in Fig. 5, the values for *k_cat_* and *K*_M_ are virtually identical for WT enzyme and the R139W variant for both NADH and menadione again indicating that the single amino acid replacement does not affect the kinetic properties.

### Structural studies: X-ray crystallography, NMR-spectroscopy and partial proteolysis

**Fig. 6:**
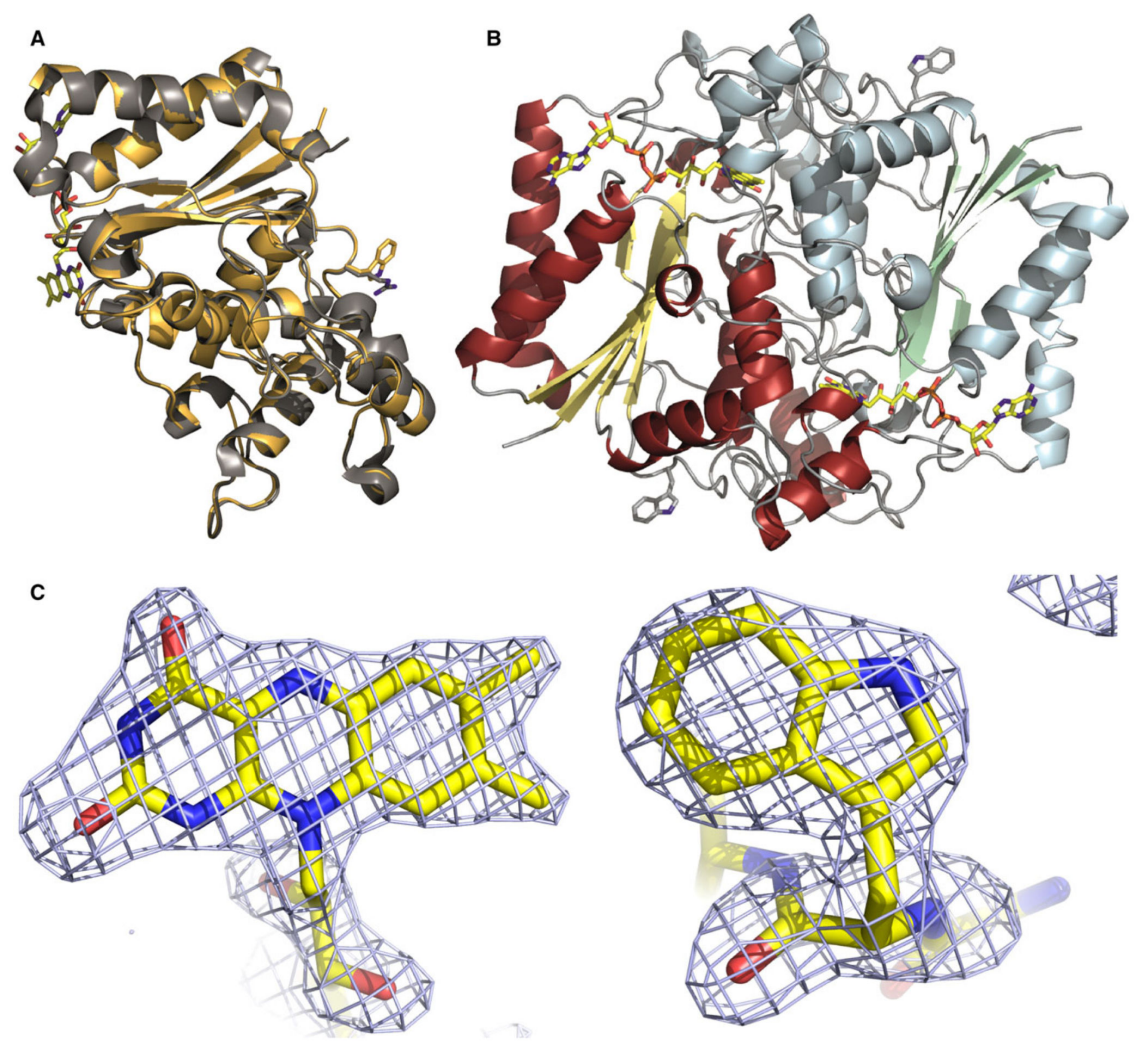
Crystal structure of NQO1 R139W. Panel A: Cartoon model of the superposition of NQO1 and NQO1 R139W with the arginine and tryptophan residue (right) and the FAD (left) shown as a stick model. Panel B: Cartoon model of the NQO1 R139W homodimer with the tryptophan residue and the FAD shown as a stick model. Panel C: Fo − Fc omit electron density contoured at 3σ for the isoalloxazine moiety of FAD bound to one of the subunits (left) and for tryptophan 139 (right).

To gain further insight into the structural properties of the R139W variant, the crystal structure of this protein was solved (see Experimental procedures and Table 4). The structure was determined to 2.1 Å and contains four protein chains in the asymmetric unit. These crystallographically independent molecules are very similar to each other, indicated by an average root-mean-square-deviation (rmsd) of 0.14 Å for a superposition of (on average) 224 out of 271 Cα-atoms. The subunit structure of the R139W variant is also virtually identical to the wild-type structure with the exception of the amino acid replacement at position 139 (Fig. 6). The respective average rmsd in this case is 0.18 Å for a superposition of 230 out of 271 Cα-atoms.

**Fig. 7:**
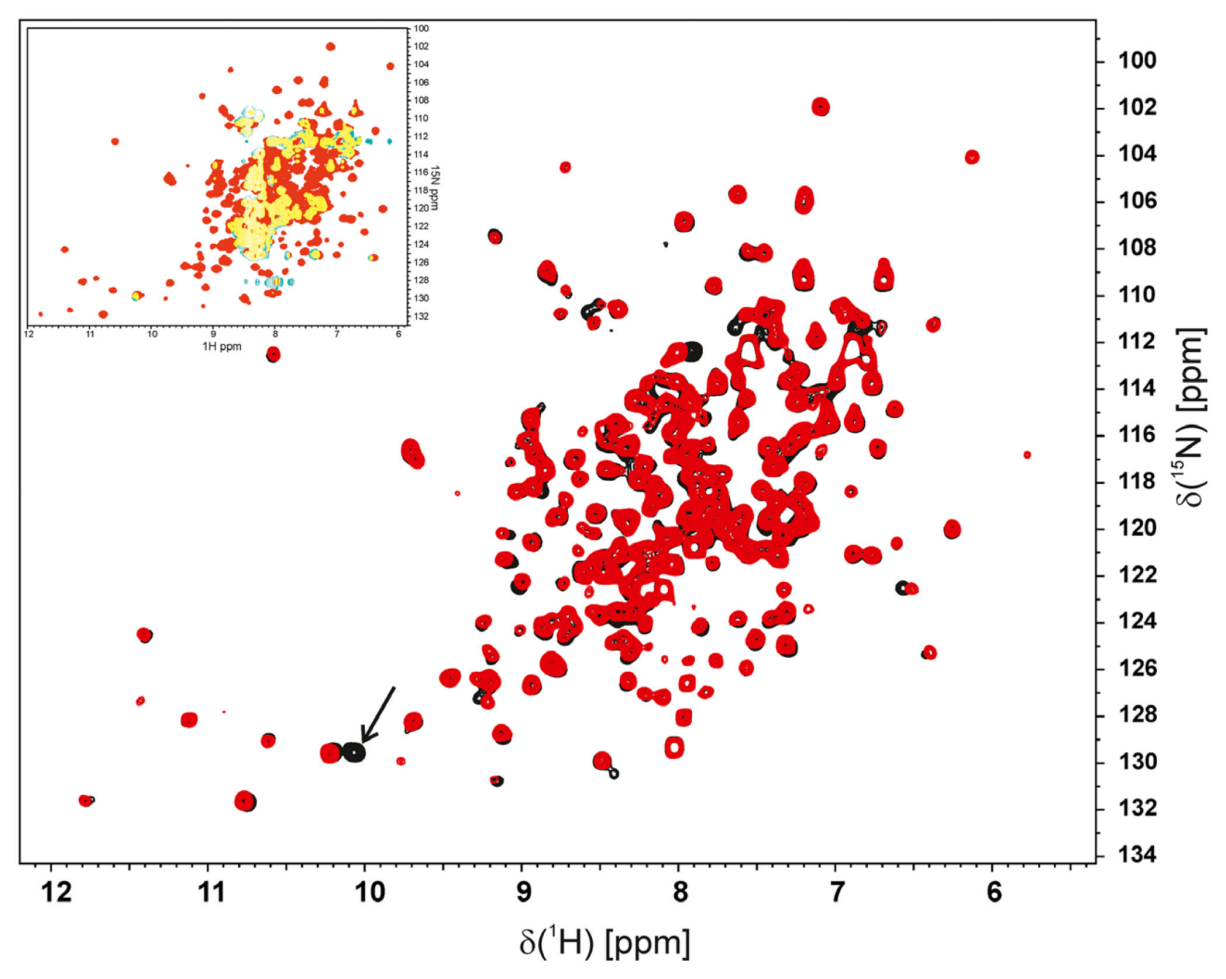
2D ^1^H-^15^N HSQC spectra. Overlay of the 2D ^1^H-^15^N HSQC spectrum of NQO1 R139W (black) and wild-type (red). An additional tryptophan side chain NH signal is visible in the R139W variant and indicated by an arrow. All other signals are almost identical in the HSQC spectrum of the R139W variant and wild-type, respectively, indicative of a very similar structure. A few minor shift differences result from residues close to residue 139. The insert in the upper left corner shows an overlay of the NMR spectra of NQO1 WT (red) and NQO1 P187S (green) where overlapping areas are marked yellow.

Recently, it was shown for the NQO1 P187S variant that despite adopting the same structure in the crystal it behaved very differently in solution as evidenced by 2D HSQC NMR-spectroscopy [14] (Fig. 7, insert). Thus, the R139W variant was also analysed using this technique. As shown in Fig. 7, the 2D HSQC spectra of NQO1 (red) and NQO1 R139W (black) are again nearly identical with the exception of an additional signal found in the region typical for a nitrogen of the indole ring in tryptophan side chains (marked by an arrow in Fig. 7). Minor shifts observed for a few signals are typical for a single amino acid exchange. Identical line-widths also indicate that the flexibilities of the two proteins are essentially unchanged.

**Fig. 8:**
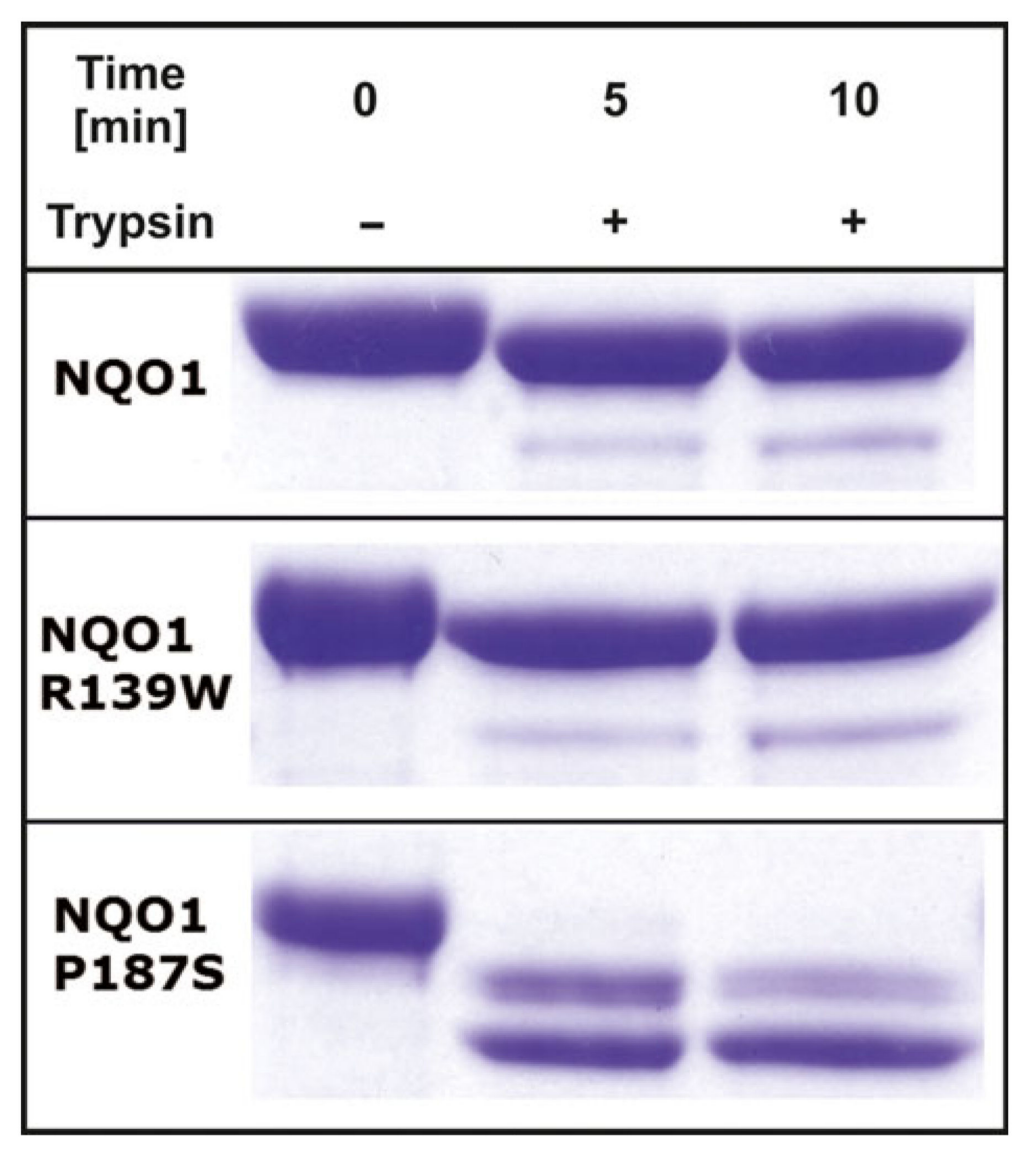
Trypsin digestion of different NQO1 variants. Comparison of Trypsin digestion of three NQO1 variants without addition of Trypsin (0 min) and two measurements after addition of Trypsin (5 and 10 min).

Recently, we also demonstrated that limited tryptic digestion can be used to monitor the structural flexibility of NQO1 variants, i.e. the unstable and partially unfolded P189S variant. In the case of the R139W variant, no difference in the digestion pattern compared to WT was detectable in agreement with the NMR-spectroscopic results (Fig. 8).

## Discussion

NQO1 constitutes an important enzyme of the cellular defence system and plays a central role in the activation of quinone-based chemotherapeutics. The occurrence of genetic variants in the human population necessitates the proper evaluation of the biochemical properties of the resulting protein variants. Previous studies on the P187S protein variant (encoded by NQO1*2) demonstrated that this single amino acid exchange causes strong destabilization of the tertiary structure leading to a substantial loss of function [14,17]. Astonishingly, it could be demonstrated that the variant adopts a very similar crystal structure, while in solution the protein is present largely in an unfolded state [14]. This very unusual and unexpected behaviour of the P187S variant prompted us to initiate a parallel study on the R139W variant caused by a single nucleotide transition in the *nqo1* gene (NQO1*3).

Initial analysis of the recombinant R139W variant by UV/visible absorption spectroscopy indicated that the affinity of the FAD cofactor as well as the nature of the cofactor binding site were not or only marginally affected by the arginine to tryptophan replacement (Fig. 1). Further studies by ITC intended to obtain dissociation constants for FAD binding revealed that the apoproteins of both wild-type and the R139W variant precipitated during the experiment, probably due to the damaging effect of shearing forces exerted by constant mixing in the sample cell. As a consequence, this particular experimental setup resulted in nonreproducible and erroneous results leading to artefacts for the stoichiometry as well as binding affinities. SAXS measurements showed that the apoprotein is not as compact as the holoprotein but still forms a dimer (Fig. 4). This may result in a lowered stability against shearing forces as well as in a lowered melting point (Table 1). Similar experiments were recently reported for wild-type NQO1 as well as the P187S and R139W variants and a sequential two site binding model was assumed to fit the data despite the fact that there is neither biochemical nor structural evidence that the two observed binding sites in the homodimeric protein are interdependent or different [17,23]. Depending on the experimental setup, the ratio of the two assumed binding sites was variable rendering the sequential binding site mode unlikely. Importantly, our ITC studies clearly demonstrate that these experimental artefacts can be avoided by simply reversing the order of the titration leading to reproducible data that is in accordance with the biochemical and structural background. We believe that this observation may also have implications for other biochemical systems investigated by ITC where one binding partner (in most cases, this will be the macromolecule rather than the small ligand) is unstable under the experimental conditions. In the case of flavoproteins, it is well-known that apoproteins are much less stable than the holoproteins in part due to the damaging effects required to prepare the apoprotein as well as the intrinsic destabilization of the overall protein structure due to depletion of the flavin prosthetic group (mostly FMN or FAD) [24].

The detailed biochemical and structural analysis of the R139W variant revealed only minor differences in comparison with wild-type NQO1. Similarly, presteady state measurements yielded almost identical bimolecular rate constants for both enzyme variants (Table 2). The reductive half reaction was extremely fast making it impossible to determine a reliable dissociation constant of NAD(P)H in our experimental setup. This indicates a low affinity of the pyridine nucleotides resulting in high *K*_D_ values as was already shown for the rat liver NQO1 [25]. Also, steady state measurements with the two enzyme variants produced comparable results. Interestingly, our measurements demonstrated that turnover hyperbolically increased with higher concentrations of NADH of up to 10 mm (!) suggesting that a canonical Michaelis-complex is not formed. This is in stark contrast to previous studies that reported *K*_M_ values in the order of about 50 to 300 μm. However, these studies have only covered a very low concentration range of NADH and thus failed to recognize the nonclassical behaviour of the enzyme [17,26]. These findings from presteady state and the steady state kinetic measurements indicate that NADH rapidly reduces the FAD cofactor of NQO1 without prior formation of a Michaelis-complex.

While the crystal structure of the previously studied variant NQO1 P187S was similar to the wild-type structure the NMR measurements revealed considerable movements of the residues [14]. In the case of the NQO1 R139W variant, we found that the crystal structure as well as the solution structure, as evidenced by NMR-spectroscopy, is virtually identical to the wild-type protein (Fig. 7). Self-association of the R139W variant caused by the higher hydrophobicity could not be observed in size exclusion chromatography. Nevertheless, we cannot exclude that under cellular conditions self-association may take place or the surface changes result in altered interaction with other proteins like the tumour suppressor p53. Tryptic digestion shows a comparable stability of NQO1 WT and NQO1 R139W in contrast to the faster degradation of NQO1 P187S as was already shown and confirmed in previous studies [14,17]. The NQO1 R139W variant only slightly differentiates from the NQO1 WT concerning FAD affinity (ca. 2.5 times weaker binding) and thermostability, by ca. 2 °C. Thus, it can be safely concluded that the expression of this variant in humans has no adverse effect on the level of NQO1 activity. Therefore, the observed effects are most probably primarily caused by erroneous splicing of the premature mRNA, leading to the loss of exon 4 and thus reducing the amount of correctly spliced NQO1 in the cell [11].

## Experimental procedures

### Reagents

All chemicals and reagents were of the highest purity commercially available from Sigma-Aldrich (St. Louis, MO, USA) and Merck (Darmstadt, Germany). Ni-NTA-agarose columns were obtained from GE Healthcare (Little Chalfont, UK).

### Molecular cloning of nqo1, protein expression and purification

The cloning of NQO1 and the generation of the NQO1 R139W variant as well as the expression and purification was carried out according to the already described procedure from Lienhart and Gudipati *et al.* [14]. The wild-type gene of NQO1 in a pET28a vector was modified with the Quick Change II XL Site-Directed Mutagenesis Kit (Agilent, Santa Clara, CA, USA) according to the provided manual with gene specific primers from Eurofins (Luxembourg).

### Apoprotein preparation and UV/Vis absorption difference titration

Apoprotein preparation and difference titration spectra were conducted as described by Lienhart and Gudipati *et al.* [14]. The measurements were made with a Specord 200 plus spectrophotometer (Analytik Jena, Jena, Germany) at 25 °C in Tandem cuvettes (Hellma Analytic, Müllheim, Germany). 800 μL of NQO1 R139W (45 μm) in the sample cell was titrated with 0–42 μL (in 2 μL intervals) and 52 μL and 62 μL in (10 μL intervals) of a FAD stock solution (1 mm). At the same time the same volume of FAD as in the sample cell was added to the buffer chamber in the reference cell and the same amount of buffer was also added to the protein chamber in the reference cell to adjust the volumes of cells. For analysis, the sum of the absolute values at 436 nm and 455 nm were plotted against the FAD/protein molar ratio.

### Small-angle X-ray scattering

SAXS data for solutions of the FAD-free and bound forms of wild-type NQO1 were recorded with an in-house SAXS instrument (SAXSspace, Anton Paar, Graz, Austria) equipped with a Kratky camera, a sealed X-ray tube source and a one-dimensional Mythen2 R 1k hybrid photon coupling detector (Dectris, Baden-Daettwil, Switzerland). The scattering patterns were measured with a 60-min exposure time (20 frames, each 3 min) with a solute concentration of 300 μm. Radiation damage was excluded on the basis of a comparison of individual frames of the 60-min exposures, wherein no changes were detected. A range of momentum transfer of 0.010 < s < 0.63 Å^−1^ was covered (s = 4π sin(θ)/λ, where 2θ is the scattering angle, and λ is the X-ray wavelength, in this case 1.5 Å.

All SAXS data were analysed with the atsas package (version 2.8, Hamburg, Germany). The data were processed with saxsquant (version 3.9) and desmeared with GNOM [27]. The forward scattering (I(0)), the radius of gyration, (*R*_g_), the maximum dimension (*D*_max_) and the interatomic distance distribution function (P(r)) were computed with GNOM [27]. The masses of the solutes were evaluated based on their Porod volume.

### Isothermal titration microcalorimetry

A VP-ITC system (MicroCal, GE Healthcare, Little Chalfont, UK) was used for calorimetric determination of the dissociation constants for FAD. The experiments were performed at 10 °C or 25 °C in 50 mm HEPES, pH 7.0 buffer or 50 mm sodium phosphate buffer with 150 mm NaCl, pH 7.0. The solutions were degassed before measurements. The titration experiments were performed with either apoprotein solution or FAD solution in the syringe and in each case the other solution in the sample cell. The concentrations of FAD and the apoprotein (concentration of NQO1 protomers) were determined spectrophotometrically. The first measurement point is rejected while the remaining data points were analysed assuming a single site or a two site binding model with origin version 7.0 (MicroCal) for ITC data analysis [14]. To remove aggregated protein, solutions were centrifuged at 21.130 ***g*** for 20 min at 22 °C.

### Steady state kinetics

Steady state parameters for NQO1 WT and NQO1 R139W were determined using a Specord 200 plus spectrophotometer (Analytik Jena, Jena, Germany) at 25 °C. NADH was used as a electron donor and menadione as a electron acceptor for the assays. The concentrations of all components were determined spectrophotometrically. For the assay with variation of NADH, the reaction mixture contained 2.5 nm WT NQO1 or the R139W variant, 200 μm menadione (ε_333 nm_ = 2450 m^−1^·cm^−1^, dissolved in ethanol, final concentration in the cuvette 1% v/v) and 1–10 mm NADH (ε_340 nm_ = 6220 m^−1^·cm^−1^) in 50 mm HEPES containing 150 mm NaCl, pH 7.0 and for the variation of menadione 2 nm NQO1 WT or NQO1 R139W, 10 mm NADH, 10–160 μm menadione (dissolved in ethanol, final concentration in the cuvette 1% v/v) in 50 mm HEPES containing 150 mm NaCl, pH 7.0. The reaction mixtures were incubated for 3 min at 25 °C and then the reaction was initiated by addition of the enzyme and the decrease in absorption of NADH was measured at 400 nm due to the high concentrations of NADH that were needed. In the case of measurements as a function of NADH concentration, the slope corresponding to the first 60 s was used for the analysis, whereas in the case of menadione variation only 10 s was used for analysis due to the fast reaction. The kinetic parameters were determined using the kaleida-graph software (Synergy Software, Reading, PA, USA).

### Transient kinetics

The rates of the reductive half reactions were determined using a Hi-Tech (SF-61DX2) stopped-flow device (TgK Scientific Limited, Bradford-on-Avon, UK), placed in a glovebox from Belle Technology (Weymouth, UK), at 4 °C. Buffer were first flushed with nitrogen and thereafter incubated in the glove box. In the same way, enzyme and substrate solutions were deoxygenated in the glove box and diluted to the desired concentration. During the experiments, enzyme was rapidly mixed with substrate and reduction in the FAD cofactor was measured by monitoring changes at 455 nm with a photomultiplier detector (PM-61s, TgK Scientific Limited, Bradford-on-Avon, UK). For these measurements, 40 μm protein was mixed with 50–2500 μm NADH or NADPH in 50 mm HEPES buffer containing 50 mm NaCl at pH 7.0. Initial rates were analysed with a hyperbolic function using the kinetic studio software (TgK Scientific).

### Crystallization and structure determination of NQO1 R139W

NQO1 R139W at 6.1 mg·mL^−1^ in 50 mm HEPES (pH 7.5) was crystallized by the microbatch method in a precipitating solution containing 200 mm Li_2_SO_4_, 100 mm BisTris (pH 6.5), 25% w/v PEG 3350 (Hampton Research Index Screen, condition 75), and incubated at 289 K. The total drop volume was 1 μL, with equal amounts of protein and precipitant solution. Yellow crystals grew to full size (~ 100 μm) within 2 months. Crystals were harvested from their mother liquor with CryoLoops™ (Hampton Research), and flash-cooled in liquid nitrogen.

A complete diffraction dataset was collected up to 2.09 Å resolution from a single triclinic crystal (space group *P*1) at the Swiss Light Source (SLS) of the Paul Scherrer Institute in Villigen, Switzerland (beamline X06DA). The data were processed using the program xds [28]. The calculated Matthews coefficient [29] indicated the presence of four molecules per asymmetric unit. The structure was determined by molecular replacement using the program Phaser [30] and the wild-type structure of NQO1 (PDB code: 1QBG) as search template.

*R*_free_ values were computed from 5% randomly chosen reflections, which were not used during refinement [31]. Structure refinement and model rebuilding were carried out with the programs phenix [32] and COOT [33,34] by alternating real-space fitting against σ_A_-weighted 2Fo − Fc and Fo − Fc electron density maps and least square optimizations. Validation of the structure was carried out with the program molprobity [35] yielding a Ramachandran plot with 97.0% of the residues in favoured regions, 3.0% in allowed and none in disallowed regions. Prediction of the biologically active form of NQO1 R137W was done using the PISA server [36]. Figures were created using the program pymol (http://www.pymol.org).

The final model was refined to *R* = 16.9% and *R*_free_ = 20.1%. Details of the data reduction and structure refinement are listed in Table 4.

### Limited proteolysis

NQO1 and NQO1 R139W (30 μm in 50 mm HEPES and 150 mm NaCl buffer at pH 7.5) were partially digested with trypsin (Promega, Madison, WI, USA) with a final concentration of 2 μg·mL^−1^ at 37 °C. The reaction was stopped after 5 and 10 min by addition of SDS sample buffer to aliquots of the reaction mixture and immediately boiled at 95 °C for 10 min. The samples were analysed by SDS/PAGE with precast gradient gels (Thermo Scientific, Waltham, MA, USA) (Fig. 8) [14,17,23,37].

### ^15^N-Labelling of NQO1 and NQO1 R139W

Minimal medium containing 6.8 g·L^−1^ Na_2_HPO_4_, 3 g·L^−1^ KH_2_PO_4_, 0.5 g·L^−1^ NaCl, 1 g·L^−1^ ^15^NH_4_Cl, 3 g·L^−1^ glucose, 1 μg·L^−1^ biotin, 1 μg·L^−1^ thiamin, 50 μg·mL^−1^ kanamycin and 1 mL 1000x microsalts [150 mm CaCl_2_, 20 mm, FeCl_3_, 50 mm H_3_BO_3_, 150 μm CoCl_2_, 800 μm CuCl_2_, 1.5 mm ZnCl_2_, 15 μm (NH_4_)_6_Mo_7_O_24_·4H_2_O] was used for protein expression as described in Lienhart and Gudipati *et al.* [14].

### NMR spectroscopy

All NMR experiments were carried out with a Bruker Avance III 700 MHz spectrometer using a cryogenically cooled 5 mm TXI probe with z-axis gradients at 298 K. Samples containing between 20–40 mg·mL^−1^ NQO1 or NQO1 R139W in 50 mm HEPES, pH 6.5 in 90% H_2_O and 10% D_2_O were used. For the ^1^H-^15^N HSQC spectra, data matrices of 2048 × 160 points were acquired and zero filled to 4k × 256 points prior to Fourier transformation. Sixty degree phase shifted squared sine bell window functions were applied in both dimensions [14].

### Thermal stability

The melting points were determined with a CFX Connect™ Real-Time PCR Detection System (Bio-Rad Laboratories, Inc., Hercules, CA, USA) by detecting the fluorescence change of SYPRO^®^ Orange Protein Gel Stain (1:5000) or the fluorescence change caused by the release of the FAD cofactor during heating [38]. The tested proteins (100 μm for FAD and 10 μm for SYPRO^®^ Orange, measured in duplicates) have been dialysed in water over night and mixed with concentrated buffer and salt solutions to obtain all tested conditions.

## Figures and Tables

**Table 1:** Thermostability measurements. Melting points were determined with a CFX Connect™ Real-Time PCR Detection System under different buffer and salt conditions of NQO1 and NQO1 R139W with FAD or SYPRO^®^ Orange as fluorescent reporter. Melting points are given in °C (values given are the average of two independent measurements).

Buffer	NQO1	NQO1 R139W
50 mm potassium phosphate, pH 7	54.0	52.0
50 mm sodium phosphate, pH 7	54.0	52.0
50 mm Tris/Cl, pH 7	54.5	52.5
50 mm HEPES, pH 7	56.0	54.0
Holoprotein in 50 mm HEPES, pH 7 + SYPRO^®^ Orange	52.8	51.2
Apoprotein in 50 mm HEPES, pH 7 + SYPRO^®^ Orange	50.8	48.5

**Table 2:** Reductive half reaction. Bimolecular rate constants (with standard deviations) of NQO1 WT and NQO1 R139W with NADH or NADPH as reducing agent.

Protein	NADH *k*_red_ (m^−1^·s^−1^)	NADPH *k*_red_ (m^−1^·s^−1^)
NQO1 WT	3.9 × 10^6^ ± 0.4 × 10^6^	7.3 × 10^6^ ± 1.4 × 10^6^
NQO1 R139W	4.6 × 10^6^ ± 0.2 × 10^6^	8.2 × 10^6^ ± 0.2 × 10^6^

**Table 3:** Steady state kinetic parameters for NQO1 and NQO1 R139W. Kinetic parameters with standard deviations determined for NQO1 and NQO1 R139W, using NADH as electron donor and menadione as electron acceptor.

	NADH			Menadione		
		
	*k*_cat,app_ (s^−1^)	*K*_M,app_ (mm)	*k*_cat,app_/*K*_M,app_ (mm^−1^.s^−1^)	*k*_cat_ (s^−1^)	*K*_M_(μm)	*k*_cat_/*K*_M_(μm^−1^.s^–1^)
NQO1 WT	1590 ± 52	3.22 ± 0.27	494 ± 44	1830 ± 82	11.6 ± 2.2	158 ± 31
NQO1 R139W	1580 ± 73	3.31 ± 0.38	478 ± 60	1730 ± 100	10.6 ± 2.8	162 ± 44	

**Table 4:** Data collection and refinement statistics.

	NQO1 R139W
Data collection	
X-ray source	SLS-X06DA
Wavelength (Å)	1.0
Temperature	100 K
Space group	*P*1
Cell dimensions	
*a*, *b*, *c* (Å)	54.61, 59.93, 99.83
α, β, γ (°)	100.37, 92.85, 90.22
Resolution (Å)^a^	49.03–2.09 (2.17–2.09)
Total reflections	194 450 (16 603)
Unique reflections	67 587 (5937)
Multiplicity^a^	2.9 (2.8)
Completeness (%)^a^	97.1 (86.89)
*R*_meas_	0.093 (0.339)
*R*_merge_	0.076 (0.296)
<I/σ_I_>^a^	12.27 (4.1)
CC_1/2_^a^	0.995 (0.906)
CC^a^	0.999 (0.975)
Refinement	
Resolution (Å)	49.03–2.09
*R*_work_/*R*_free_	0.1693/0.2013
No. of atoms	
Protein	8659
Cofactor/ligands	240
Water	1052
Mean B-factors (Å^2^)	
Protein	23.40
Cofactor/ligands	26.60
Water	32.30
All atoms	24.40
R.m.s. deviations	
Bond lengths (Å)	0.004
Bond angles (°)	0.95
Ramachandran outliers (%)	0
PDB-entry	5A4K

a Values in parentheses are for the highest resolution shell.

## Abbreviations

ITC: isothermal titration microcalorimetry
NQO1: NAD(P)H:quinone oxidoreductase 1
PDB: protein data bank
SAXS: small-angle X-ray scattering
SNP: single-nucleotide polymorphism
WT: wild-type
